# Complications after hip fracture surgery: are they preventable?

**DOI:** 10.1007/s00068-017-0826-2

**Published:** 2017-08-09

**Authors:** E. R. Flikweert, K. W. Wendt, R. L. Diercks, G. J. Izaks, D. Landsheer, M. Stevens, I. H. F. Reininga

**Affiliations:** 1 Department of Surgery-Traumatology, University Medical Center Groningen, UMCG, University of Groningen, P.O. Box 30001, 9700 RB Groningen, The Netherlands; 2 Department of Orthopedic Surgery, University Medical Center Groningen, University of Groningen, Groningen, The Netherlands; 3 University Center for Geriatric Medicine, University Medical Center Groningen, University of Groningen, Groningen, The Netherlands

**Keywords:** Hip fracture, Frail elderly, Complications

## Abstract

**Purpose:**

Surgery for hip fractures is frequently followed by complications that hinder the rehabilitation of patients. The aim of this study was to describe the incidence rate and type of complications, including mortality, after hip fracture surgery, and to identify the risk factors of these complications that may be amenable to prevention.

**Methods:**

Prospective cohort study of all consecutive patients aged ≥60 treated for a hip fracture at University Medical Center Groningen between July 2009 and June 2013. All patients were treated in a comprehensive multidisciplinary care pathway. Logistic regression analyses were used to investigate which variables were significant risk factors for the occurrence of complications. Additional analyses were conducted to investigate whether the independent variables were significant risk factors for several specific complications and mortality.

**Results:**

The study population consisted of 479 patients with a mean age of 78.4 (SD 9.5) years; 33% were men. The overall complication rate was 75%. Delirium was the complication seen most frequently (19%); the incidence of surgical complications was 9%. Most risk factors for complications were not preventable (high comorbidity rate, high age and dependent living situation). However, general anesthesia (OR 1.51; 95% CI 0.97–2.35) and delay in surgery (OR 3.16; 95% CI 1.43–6.97) may be risk factors that can potentially be prevented. Overall, the mortality risk was not higher in patients with a complication, but delirium and pneumonia were risk factors for mortality.

**Conclusion:**

The overall complication rate after hip fracture surgery was high. Only few complications were potentially preventable.

## Purpose

Hip fractures are frequently encountered; the estimated worldwide incidence for the year 2000 is 1.6 million cases [1]. Most patients with a hip fracture are older and have multiple comorbidities. Most of them are female and have experienced an indoor fall [2]. Despite advances in surgical and anesthetic techniques over time, morbidity and mortality after hip fracture surgery remain high. From an economic perspective and of course also for future patients lowering such morbidity and mortality rates is an important aim.

A wide range of complications are observed after hip fracture surgery. Only a small part of them is related to the surgical procedure, such as a wound infection and loss of reduction. However, because the described population consists of mostly frail elderly persons, the most commonly encountered complications are non-surgical, such as delirium, pneumonia, heart failure and pressure ulcers [3–5]. There is a shortage of literature describing risk factors for complications after hip fracture surgery. Comorbidity and delay in surgery are reported risk factors [6].

The 1-year mortality rate after hip fracture surgery of 12–33% is high, up to eight times the normal mortality rate for people aged 80 [3, 7, 8]. Risk factors for mortality after hip fracture surgery have been described in the literature. Examples of these risk factors are advanced age, male gender, living in a nursing home, poor preoperative walking capacity and high ASA score [9].

It is important to identify risk factors for complications and mortality after hip fracture surgery, especially those that can be influenced, to ameliorate outcomes and improve postoperative functioning in daily life. In addition, a reduction in complication rates will reduce costs for society. Hence, the aim of this study was to describe the incidence rate and type of complications and mortality after hip fracture surgery as well as to identify existing and preventable risk factors for both.

## Methods

### Design

A prospective cohort study was conducted at the Departments of Trauma Surgery and Orthopedic Surgery of University Medical Center Groningen (UMCG) in the Netherlands. Informed consent was obtained from all patients and the study was approved by the Medical Ethical Committee of UMCG (METc 2011/164). The study was conducted in accordance with the Declaration of Helsinki.

### Patients

All patients aged 60 years or older with a hip fracture treated at UMCG between 1 July 2009 and 1 May 2013 were included in this study. Patients were excluded if they had multiple injuries caused by high energetic trauma. A hip fracture was defined as a femoral neck fracture (dislocated or not dislocated) or a trochanteric fracture [subdivided into type A1, A2 or A3 according to the Arbeitsgemeinschaft für Osteosynthesefragen (AO) comprehensive classification]. In 2009, a comprehensive care pathway for the treatment of hip fractures was implemented at UMCG. The care pathway has been described before [10]. This comprehensive multidisciplinary care pathway was developed to include all elements of care in the trajectory, from arrival in the emergency room to the moment of discharge from the rehabilitation unit of the nursing home. This operative protocol for hip fractures at UMCG is based on the Dutch surgical guideline for hip fractures [11].

### Data

The electronic files of the included patients were searched for complications occurring from admittance up to the last outpatient visit 6 months after surgery. Registered baseline characteristics were demographic information, medical history, fracture classification, trauma mechanism, living situation, American Society of Anesthesiologists (ASA) classification and baseline blood values (hemoglobin and electrolytes). The Charlson comorbidity index (CCI) was calculated. This score is a method for estimating the risk of death based on comorbidities [12]. To calculate the score, 22 comorbid conditions are assigned a score of 1, 2, 3 or 6, depending on the risk of dying associated with each one. The index is the sum of the scores [12]. During hospitalization type of implant, waiting time to surgery, operation time, type of anesthesia, preoperative fasting time, length of hospital stay and blood values at discharge were taken from the electronic hospital registration system. Clinical complications and side effects were registered. All complications were also registered up to 6 months postoperatively; the mortality rate was followed up to 1 year after the operation.

Complications were divided into minor and major complications. Examples of minor complications are: hematoma, urine retention, electrolyte disturbances and leg length discrepancy. Examples of major complications are: all reoperations, delirium, pneumonia, cardiac and neurological complications, and pressure ulcers.

### Statistical analysis

Descriptive statistics were used to describe the characteristics of the study population at baseline and the number and type of complications after surgery. Logistic regression analysis was used to investigate which variables were significant risk factors for the occurrence of perioperative complications. In this analysis, the dependent variable was complication (yes/no) and the independent variables were age, gender, fracture type, CCI, pre-fracture living situation, >1 day delay to first procedure (yes/no) and type of anesthesia (spinal vs. general). For the analysis patients were divided into four age groups (60–65, 65–75, 75–85, >85), four groups of CCI (0, 1, 2, ≥3), and four groups of living situation (living independently, independently with others, in an assisted living facility, in a nursing home).

Additional analyses were conducted to investigate whether the independent variables were significant risk factors for several specific complications (delirium, congestive heart failure and pneumonia). In the logistic regression analyses, the independent variables were first introduced separately in univariate models, after which multivariate models were constructed with the variables with *p* < 0.20 using a stepwise forward selection method. Variables with *p* ≤ 0.10 were retained in the final multivariate models. Odds ratios (OR) with the corresponding 95% confidence intervals (CI) were calculated for each independent variable.

Cox proportional hazards regression analyses were performed to estimate the association between several independent variables (age, gender, fracture type, complication, etc.) and 1-year mortality. First, univariate regression models were created, after which the variables with *p* < 0.20 were eligible to be included in the multivariate regression models. A stepwise forward selection method was used. Variables with *p* ≤ 0.10 were retained in the final multivariate models. Hazard ratios (HR) with the corresponding 95% CI were calculated. All statistical analyses were performed using SPSS Statistics for Windows (Version 22.0, Armonk, NY: IBM Corp.). *p* < 0.05 was considered to indicate statistical significance.

## Results

The study cohort consisted of 479 patients. Baseline characteristics are presented in Table 1. Most patients were elderly women with a mean age of 78.4 (SD 9.5) years, with one or more comorbidities and a median ASA classification of 3 [interquartile range (IQR) 1–4], and who lived independently (40%). The first surgical procedure for the vast majority of patients took place within the first day after admission (*N* = 435; 91%). Median (IQR) operation time was 92 (30–310) min. General anesthesia was used in 310 patients (65%) and spinal anesthesia in 169 patients (35%). Mean hospital stay was 8.8 days (SD 7.7).

**Table 1:** Baseline characteristics of the study population (*N* = 479)

Age (years)^a^	78.4 (9.5)
Age per category (years)^b^	
60–65	45 (10)
66–75	130 (27)
76–85	155 (32)
≥86	149 (31)
Gender^b^	
Male	158 (33)
Female	321 (67)
ASA classification^b^	
1	28 (6)
2	173 (36)
3	250 (52)
4	28 (6)
Charlson comorbidity index^b^	
0	140 (29)
1	103 (22)
2	108 (22)
≥3	128 (27)
Prefracture living situation^b^	
Independently	181 (38)
Independently, with help of others	129 (27)
Assisted living facility	72 (15)
Nursing home	68 (14)
Unknown	29 (6)
Fracture type^b^	
A1	55 (12)
A2	107 (22)
A3	57 (12)
Femoral neck, undisplaced	55 (12)
Femoral neck, displaced	205 (42)
Type of anesthesia^b^	
Spinal	169 (35)
General	310 (65)
Days to first procedure^b^	
<2 day	435 (91)
≥2 day	44 (9)
Length of hospital stay (days)^a^	8.8 (7.7)

^a^ Data presented as mean (SD)

^b^ Data presented as *N* (%)

### Incidence of complications

A total of 359 patients (75%) suffered one or more complications during the 6 months of follow-up; 210 patients (44%) suffered multiple complications (Table 2). Only 119 patients (25%) did not have any negative side effects from the treatment. Of all complications, 173 (48%) were minor and 186 (52%) were classified as major. The three most frequently encountered medical complications were delirium (*N* = 98, 20%), pneumonia (*N* = 47, 10%) and congestive heart failure (*N* = 25, 5%). Pressure ulcers were registered in five patients (1%). Complications related to the operative procedure were seen in 13% of the patients (total wound and implant problems *N* = 63). Reoperation was performed in 52 patients (11%) during the follow-up period. Eighty patients (16%) only had complications that were registered during outpatient visits; most seen complications at this time were leg length difference and persistent pain. After 1 year, 129 patients (27%) had died.

**Table 2:** Incidence of complications during 6 months of follow-up after surgery for hip fracture

	*N* (%)
Number of complications	
None	119 (25)
One	150 (31)
Multiple	210 (44)
Specific complications	
Delirium	98 (20)
Pneumonia	47 (10)
Heart failure	25 (5)
Pressure ulcer	5 (1)
Wound problems	43 (9)
Surgical or implant-related	20 (4)
Only during follow-up (outpatient clinic)	80 (16)
Reoperation	52 (11)
Mortality (1 year)	129 (27)

### Risk factors of complications

A statistically significant association was found between a CCI score ≥3 and the occurrence of complications after hip fracture surgery [OR = 3.19, 95% CI 1.67–6.08, *p* < 0.001 (Table 3)]. There was no statistically significant association between other independent variables and the occurrence of complications. Patients who received general instead of spinal anesthesia did have more complications, but this difference did not reach statistical significance (OR = 1.51, 95% CI 0.97–2.35, *p* = 0.07).

**Table 3:** Risk factors for complications, logistic regression analysis

Risk factor	Regression coefficient	*p* value	OR (95% CI)
Fracture type (ref. type A1)			
A2	0.68	0.08	1.97 (0.93–4.17)
A3	0.88	0.056	2.40 (0.98–5.91)
Neck, undisplaced	0.12	0.78	1.12 (0.50–2.54)
Neck, displaced	0.54	0.11	1.71 (0.88–3.30)
Charlson comorbidity index (ref. 0)			
1	−0.04	0.88	0.96 (0.55–1.68)
2	0.41	0.17	1.50 (0.84–2.68)
≥3	1.17	<0.001	3.19 (1.67–6.08)
Male sex	−0.40	0.08	0.67 (0.43–1.06)
General anesthesia (ref. spinal anesthesia)	0.41	0.07	1.51 (0.97–2.35)

*OR* odds ratio, *CI* confidence interval

Delirium was the most frequently observed complication. Not living independently, higher age and delay of surgery of more than 1 day were all statistically significant risk factors for getting delirium (Table 4). There was only one independent risk factor for congestive heart failure: CCI ≥ 3 (OR = 16.49, 95% CI 2.13–127.57, *p* = 0.007). For postoperative pneumonia, the relationship with CCI was borderline significant (OR = 2.18, 95% CI 0.97–4.90, *p* = 0.06). There was no statistically significant association between postoperative pneumonia and the other variables.

**Table 4:** Risk factors for delirium, logistic regression analysis

Risk factor	Regression coefficient	*p* value	OR (95% CI)
Charlson comorbidity index (ref. 0)			
1	−0.03	0.94	0.97 (0.44–2.15)
2	0.33	0.40	1.39 (0.65–2.94)
≥3	0.63	0.08	1.88 (0.94–3.75)
Prefracture living situation (ref. living independently)			
Independently, with help of others	−0.11	0.76	0.90 (0.44–1.81)
Assisted living facility	0.69	0.05	1.99 (1.00–4.00)
Nursing home	0.87	0.02	2.38 (1.18–4.81)
Age (ref. 60–65)			
66–75	0.29	0.66	1.33 (0.37–4.77)
76–85	1.05	0.08	2.85 (0.87–9.35)
≥86	1.40	0.02	4.05 (1.23–13.33)
Male sex	0.45	0.10	1.57 (0.93–2.68)
Days to procedure >2	1.15	0.005	3.16 (1.43–6.97)

*OR* odds ratio, *CI* confidence interval

### Association between complications and mortality

Patients with a complication did not have a higher mortality rate (OR = 1.18, 95% CI 0.70–2; *p* = 0.52, Table 5). In univariate analyses, only CCI and higher age were found to be statistically significant predictors of mortality. However, when independent variables, including the various complications, were investigated in multivariate models, delirium as well as pneumonia increased mortality risk after hip fracture surgery [HR = 2.20, 95% CI 1.46–3.32, *p* < 0.001; and HR = 2.05, 95% CI 1.24–3.39, *p* = 0.005, respectively (Table 6)].

**Table 5:** Risk factors for mortality, Cox proportional hazards regression analysis, univariate analysis

Risk factor	Regression coefficient	*p* value	HR (95% CI)
Complication	0.17	0.52	1.18 (0.70–2.00)
Charlson index (ref. 0)			
1	0.28	0.46	1.32 (0.63–2.79)
2	0.82	0.02	2.27 (1.18–4.83)
≥3	1.29	<0.001	3.62 (1.98–6.64)
Age (ref. 60–65)			
66–75	0.60	0.34	1.82 (0.53–6.25)
76–85	1.14	0.06	3.13 (0.96–10.18)
≥86	1.46	0.02	4.30 (1.33–13.91)
Male sex	0.36	0.08	1.44 (0.96–2.15)

*HR* hazard ratio, *CI* confidence interval

**Table 6:** Risk factors for mortality, Cox proportional hazards regression analyses, multivariate analysis

Risk factor	Regression coefficient	*p* value	HR (95% CI)
Delirium	0.79	<0.001	2.20 (1.46–3.32)
Pneumonia	0.72	0.005	2.05 (1.24–3.39)
Charlson index (ref. 0)			
1	0.23	0.55	1.26 (0.60–2.64)
2	0.79	0.02	2.21 (1.14–4.26)
≥3	1.20	<0.001	3.31 (1.81–6.07)
Age (ref. 60–65)			
66–75	0.51	0.42	1.66 (0.48–5.73)
76–85	0.95	0.12	2.59 (0.79–8.47)
≥86	1.21	0.04	3.37 (1.04–10.93)

*HR* hazard ratio, *CI* confidence interval

## Discussion

The aim of this study was to describe the complications and mortality after hip fracture surgery and to identify risk factors for both. Most remarkable outcome of this study was the high percentage of patients suffering one or more complications, namely 75%, half of which were major.

Complication rates after hip fracture surgery reported in the literature vary widely between 12.5 and 57% [3, 6, 13, 14]. Possible causes of these differences in rates are study design, time to follow-up, and definition of a complication. The complication rate in our study was higher than in the literature, maybe because in our study population all kinds of side effects (surgical, medical but also long-term) were considered complications. In addition, all electronic patient files were meticulously searched again for complications after the follow-up of 6 months to complete the prospective database. It is important to realize that only a minority of hip fracture patients will be free from side effects.

### Incidence of complications

The three most encountered medical complications were delirium, pneumonia and congestive heart failure. A delirium incidence of 20% was found in this study. The reported incidence of delirium varies widely between 4 and 53% [15]. The comprehensive care pathway followed in this study included consultation with a geriatrician. There is some evidence that delirium can be prevented or that its severity can be reduced after geriatric consultation [16]. On the other hand, a geriatrician will diagnose delirium sooner than other specialists, resulting in a higher yet more reliable incidence rate.

Pneumonia and congestive heart failure were the second and third most prevalent medical complications, with an incidence of, respectively, 10 and 5%. This is in accordance with other research [3, 17]. Possible preventive measures for these complications that were already included in the protocol for the care pathway is this study were investigation of cardiac status by the anesthesiologist, surgery on the first day after admission in supine position and early mobilization.

All surgical complications together formed 13% of the complications. Because of the heterogeneity of these complications and the small numbers of each of its different components (hematoma, infection, different implant problems), this was not further analyzed. The incidence of surgical complications matches the literature. The reported surgical site infection rate after hip fracture surgery is about 5% for orthopedic trauma surgery [18, 19]. The incidence of reoperation depends on fracture type and implant, but is reported between 7 and 38% in femoral neck fractures [20, 21].

The percentage of pressure ulcers in our study was low (1%), and much lower than reported in the literature; a recent study conducted in the US showed that about one-third of hip fracture patients developed a pressure ulcer during follow-up [22]. An explanation for the low incidence could be the special preventive measures that were taken in this study. Already on the ER patients with a hip fracture were placed on a pressure ulcer-preventive mattress, and almost all patients were operated on within one day. However, the number of pressure ulcers might have been underestimated as admission time to the hospital was short, and probably not every ulcer was reported by the nursing home physician or seen during physical examination at the outpatient clinic.

### Risk factors of complications

In this study, the only significant risk factor of developing a complication was having a high CCI (≥3). Other studies also show that having more comorbidities is the strongest risk factor for complications [3, 6]. Risk factors close to statistical significance are having an A3 type intertrochanteric fracture and getting general instead of spinal anesthesia. The A3 type intertrochanteric fracture is classified as unstable and more difficult to treat than other trochanteric fractures. Patients seem to need more analgesics [23], which might lead to more complications. Type of anesthesia is a modifiable risk factor. In this study, the number of patients that underwent general anesthesia was high (65%). Whether general or spinal anesthesia leads to fewer complications in hip fracture patients is still a matter of debate [24–27]. The recent Cochrane review did not find a difference in either technique and asked for more research [28]. Most important factor seems to be the patient himself: for some cardiac conditions general anesthesia is more safer, for other pulmonary or neurological conditions spinal anesthesia is preferred.

Another known, possibly preventable risk factor for complications is delay of surgery for more than 2 days [6]. This effect was not seen in the present study, perhaps because one part of the care pathway was to operate on all patients the morning after admission, so only for a small number of patients (9%) was the operation postponed longer than 2 days. Surgery was only postponed for strict medical reasons such as sepsis or fulminant pneumonia; in these cases, these medical reasons were considered comorbidities, not complications, as they were already present at arrival in the emergency room.

### Risk factors for delirium, pneumonia and congestive heart failure

The risk for delirium was higher in older patients and those living in a nursing home; some of these patients probably had cognitive dysfunction already (in this study population this was not measured during admittance), and these patients are known to be at risk for delirium [29]. In general, patients living in a nursing home have a lower performance status in comparison to patients living independently; mostly their CCI is higher resulting in a higher complication rate, as shown especially in delirium. Social isolation might play a role as a risk factor for delirium. However, a definitive answer cannot be given. Patients living independently may live as much or even more in social isolation as patients in nursing homes do. In the literature there is some evidence that the presence of family members in the hospital will help to reduce the incidence of delirium [30], moreover, that social isolation leads to a prolonged hospitalization [31].

The risk factors for delirium found in this study are also in line with the British NICE guidelines for delirium, with the most important risk factors attributed to older age, cognitive impairment and having a hip fracture [32]. The only preventable risk factor for delirium known in the literature is delay in surgery for more than 1 day, resulting in a prolonged period of bed rest and pain. We therefore believe it should be a goal of care pathways to operate patients with a hip fracture within a day of admission. In accordance with the recent literature [33], in our study general anesthesia was not associated with a higher risk of delirium.

Early mobilization is one of the aspects of the comprehensive care pathway. Research has shown that early mobilization is probably one of the most important measures to reduce the incidence of pneumonia [34]. This study did not show other preventable independent risk factors for pneumonia or congestive heart failure.

### Complications and mortality

The 1-year mortality rate was 27%. Although most studies report mortality rate after 3 or 4 months, this annual rate is in accordance with the literature [3, 8]. Mortality was not influenced by complications in general. The only independent risk factors for mortality were having delirium or pneumonia postoperatively. It is generally assumed that having delirium is a poor prognostic sign, and Witlox et al. showed in their meta-analysis that delirium has been proven to be an independent risk factor not only for mortality but also for institutionalization and dementia [35]. This stresses the importance of reducing the risk of delirium. Higher postoperative mortality after pneumonia has been described before [3] and is probably partly due to poor physical condition, which hampers mobilization.

## Conclusion

Complication rates after hip fracture surgery are very high, reaching 75%. Medical complications such as delirium and pneumonia are encountered more frequently than surgical complications. The 1-year mortality rate in this study was 27%, and both delirium and pneumonia were risk factors for mortality. Although most risk factors cannot be influenced, early operation and mobilization seem to be important in trying to reduce the number of complications and mortality.

## References

[1] Johnell O, Kanis JA (2006). An estimate of the worldwide prevalence and disability associated with osteoporotic fractures. Osteoporos Int.

[2] Ranhoff AH, Holvik K, Martinsen MI, Domaas K, Solheim LF (2010). Older hip fracture patients: three groups with different needs. BMC Geriatr.

[3] Roche JJ, Wenn RT, Sahota O, Moran CG (2005). Effect of comorbidities and postoperative complications on mortality after hip fracture in elderly people: prospective observational cohort study. BMJ.

[4] Kalisvaart KJ, de Jonghe JF, Bogaards MJ, Vreeswijk R, Egberts TC, Burger BJ (2005). Haloperidol prophylaxis for elderly hip-surgery patients at risk for delirium: a randomized placebo-controlled study. J Am Geriatr Soc.

[5] Hung WW, Egol KA, Zuckerman JD, Siu AL (2012). Hip fracture management: tailoring care for the older patient. J Am Med Assoc.

[6] Belmont PJ, Garcia EJ, Romano D, Bader JO, Nelson KJ, Schoenfeld AJ (2014). Risk factors for complications and in-hospital mortality following hip fractures: a study using the National Trauma Data Bank. Arch Orthop Trauma Surg.

[7] Haentjens P, Magaziner J, Colon-Emeric CS, Vanderschueren D, Milisen K, Velkeniers B (2010). Meta-analysis: excess mortality after hip fracture among older women and men. Ann Intern Med.

[8] Lopez JG, Rossell CP, Remon JH, Gomez GC, Salvado GR, Coll BS (2002). One-year outcome in older patients undergoing surgery for hip fractures. Results of a treatment protocol. Rev Orthop Traumatol..

[9] Hu F, Jiang C, Shen J, Tang P, Wang Y (2012). Preoperative predictors for mortality following hip fracture surgery: a systematic review and meta-analysis. Injury.

[10] Flikweert ER, Izaks GJ, Knobben BA, Stevens M, Wendt K (2014). The development of a comprehensive multidisciplinary care pathway for patients with a hip fracture: design and results of a clinical trial. BMC Musculoskelet Disord..

[11] Van Vugt A. Richtlijn Behandeling van de proximale femurfractuur bij de oudere mens Dutch Society of Surgery. 2008. http://www.heelkunde.nl/uploads/gt/I9/gtI9yRKuAUxcHvz1YB92sg/Behandeling-van-de-proximale-femurfractuur-bij-de-oudere-mens-2008.pdf. Accessed 12 Dec 2014.

[12] Charlson ME, Pompei P, Ales KL, MacKenzie CR (1987). A new method of classifying prognostic comorbidity in longitudinal studies: development and validation. J Chronic Dis.

[13] Leung AH, Lam TP, Cheung WH, Chan T, Sze PC, Lau T (2011). An orthogeriatric collaborative intervention program for fragility fractures: a retrospective cohort study. J Trauma.

[14] Dy CJ, Dossous PM, Ton QV, Hollenberg JP, Lorich DG, Lane JM (2012). The medical orthopaedic trauma service: an innovative multidisciplinary team model that decreases in-hospital complications in patients with hip fractures. J Orthop Trauma.

[15] Bruce AJ, Ritchie CW, Blizard R, Lai R, Raven P (2007). The incidence of delirium associated with orthopedic surgery: a meta-analytic review. Int Psychogeriatr.

[16] Deschodt M, Braes T, Flamaing J, Detroyer E, Broos P, Haentjens P (2012). Preventing delirium in older adults with recent hip fracture through multidisciplinary geriatric consultation. J Am Geriatr Soc.

[17] Brown CA, Boling J, Manson M, Owens T, Zura R (2012). Relation between prefracture characteristics and perioperative complications in the elderly adult patient with hip fracture. South Med J.

[18] Hansis M, Arens S, Wingenfeld C (1997). Rate of infection in trauma surgery. An overview based on recent German language literature. Unfallchirurg.

[19] Merrer J, Girou E, Lortat-Jacob A, Montravers P, Lucet JC (2007). Groupe de Recherche sur l’Antibioprophylaxie en Chirurgie. Surgical site infection after surgery to repair femoral neck fracture: a French multicenter retrospective study. Infect Control Hosp Epidemiol.

[20] Murphy DK, Randell T, Brennan KL, Probe RA, Brennan ML (2013). Treatment and displacement affect the reoperation rate for femoral neck fracture. Clin Orthop Relat Res.

[21] Griffin J, Anthony TL, Murphy DK, Brennan KL, Brennan ML (2016). What is the impact of age on reoperation rates for femoral neck fractures treated with internal fixation and hemiarthroplasty? A comparison of hip fracture outcomes in the very elderly population. J Orthop.

[22] Baumgarten M, Margolis DJ, Orwig DL, Shardell MD, Hawkes WG, Langenberg P (2009). Pressure ulcers in elderly patients with hip fracture across the continuum of care. J Am Geriatr Soc.

[23] Mak JC, Lattouf I, Narushevich A, Lai C, O’Rourke F, Shen Q (2011). A prospective review of hip fracture subtypes, surgical procedure, cognitive status, and analgesia use across four Australian hospitals. Geriatr Orthop Surg Rehabil.

[24] Fields AC, Dieterich JD, Buterbaugh K, Moucha CS (2015). Short-term complications in hip fracture surgery using spinal versus general anaesthesia. Injury.

[25] Parker MJ, Griffiths R (2015). General versus regional anaesthesia for hip fractures. A pilot randomised controlled trial of 322 patients. Injury.

[26] Tung YC, Hsu YH, Chang GM (2016). The effect of anesthetic type on outcomes of hip fracture surgery: a nationwide population-based study. Medicine (Baltimore).

[27] Luger TJ, Kammerlander C, Gosch M, Luger MF, Kammerlander-Knauer U, Roth T (2010). Neuroaxial versus general anaesthesia in geriatric patients for hip fracture surgery: does it matter?. Osteoporosis Int.

[28] Guay J, Parker MJ, Gajendragadkar PR, Kopp S (2016). Anaesthesia for hip fracture surgery in adults. Cochrane Database Syst Rev..

[29] Oh ES, Li M, Fafowora TM, Inouye SK, Chen CH, Rosman LM (2015). Preoperative risk factors for postoperative delirium following hip fracture repair: a systematic review. Int J Geriatr Psychiatry..

[30] Abraha I, Rimland JM, Trotta F, Pierini V, Cruz-Jentoft A, Soiza R (2016). Non-pharmacological interventions to prevent or treat delirium in older patients: clinical practice recommendations The SENATOR-ONTOP series. J Nutr Health Aging.

[31] Landeiro F, Leal J, Gray AM (2016). The impact of social isolation on delayed hospital discharges of older hip fracture patients and associated costs. Osteoporos Int.

[32] Young J, Murthy L, Westby M, Akunne A, O’Mahony R (2010). Guideline Development Group. Diagnosis, prevention, and management of delirium: summary of NICE guidance. BMJ.

[33] Hussain M, Berger M, Eckenhoff RG, Seitz DP (2014). General anesthetic and the risk of dementia in elderly patients: current insights. Clin Interv Aging.

[34] Kamel HK, Iqbal MA, Mogallapu R, Maas D, Hoffmann RG (2003). Time to ambulation after hip fracture surgery: relation to hospitalization outcomes. J Gerontol A.

[35] Witlox J, Eurelings LS, de Jonghe JF, Kalisvaart KJ, Eikelenboom P, van Gool WA (2010). Delirium in elderly patients and the risk of postdischarge mortality, institutionalization, and dementia: a meta-analysis. JAMA.

